# Conflicts of Interest in Nutrition: Categorical Thinking and the Stigma of Commercial Collaboration

**DOI:** 10.1016/j.cdnut.2024.104413

**Published:** 2024-07-17

**Authors:** David J Mela

**Affiliations:** Valkenswaard, The Netherlands

**Keywords:** commercial conflicts, financial conflicts, intellectual conflicts, declarations, management

## Abstract

There is a high level of concern about the possible influence of commercial organizations on food-related research and professional bodies, including regulatory and advisory panels. This has contributed to an increased emphasis on the declaration and management of conflicts of interest (CoI) in the reporting, evaluation, and application of research in nutrition science. However, common perceptions of CoI in nutrition, and procedures for declaring and managing these, often lack intellectual rigor and consistency. This commentary highlights 3 main issues related to CoI in nutrition, particularly the emphasis on industry-related CoI relative to other sources of conflict and bias. *1*) Considerations of CoI in nutrition are largely limited to financial or collaborative links to the food industry, disregarding other important sources of influence such as intellectual allegiances or nonindustry financial and professional incentives. *2*) Associations with industry incur *ad hominem*, often punitive stigmatization of individuals and their research, and inappropriate downgrading or exclusion of evidence. This disproportionately affects expertise in the food and agricultural sciences, in which commercial collaborations are widely encouraged. *3*) These practices and related approaches to managing CoI are applied without due consideration of the nature of the conflicts and activities involved, the qualifications of individuals, or the availability of other, objective methods and guidance for assessing research quality and risks of bias. Overall, recognition of the nature and range of CoI in nutrition and approaches to their identification and management lack consistency and balance. A singular and strict focus specifically on industry-related CoI may paradoxically exacerbate rather than mitigate imbalance and bias in the field. This commentary outlines the underlying issues and the need for more comprehensive and nuanced approaches to the assessment, reporting, and management of CoI in nutrition.

## Introduction

There is a long history of debate around the influence and management of possible conflicts of interest (CoI) in the biomedical sciences, including nutrition, and an extensive professional literature addressing the ethical, legal, and practical aspects of CoI [[1], [2], [3], [4]]. A widely applied definition of CoI is “…a set of circumstances that creates a risk that professional judgment or actions regarding a primary interest will be unduly influenced by a secondary interest” [5]. In the context of nutrition, a primary interest is typically the quality and integrity of research, professional opinions, or recommendations intended to benefit patients or public health. Secondary interests that may influence this include personal and professional relationships with other individuals or organizations and one’s own self-interests and beliefs. CoI raises concerns that secondary interests or inducements influence judgments to favor those interests. CoI also highlights a risk of bias, an inclination to favor certain viewpoints, relative weightings or interpretations of evidence, or specific policy or guidance alternatives.

In the field of nutrition, concerns around CoI pervade discussions of research generation and publication, expert advisory committees, and policy-making. It is fair to ask although whether the general understanding and management of CoI by journals, public health authorities, and the wider community are consistent, fair, and fit for purpose. Most importantly, do current approaches to identifying and managing CoI in nutrition maximize the quality, range, and balance of expertise and evidence applied to public health issues?

There are several aspects to the definition of CoI that warrant attention. First, although often focused on links to the food industry, CoI is not limited to external commercial influences. CoI may comprise a wide range of secondary interests relating to financial and other personal and professional benefits and associations, as well as deeply established intellectual loyalties and allegiances [6,7]. Second, the intent or motivation of individuals does not come into this definition; i.e., the conflict may be present whether individuals are believed to be influenced by self-interest or altruism. Third, a CoI does not *per se* indicate any violation of integrity, but highlights a situation where there is a *risk* of being influenced [8], prompting further consideration of assessment of that risk. “A CoI describes a situation in which there is a risk of bias and resulting harm, not a situation in which bias or harm necessarily occurs” [9]. Classifications using terms such as “potential,” “perceived,” “actual,” or “primary” compared with “secondary” CoI may also be used where defined for certain purposes [10,11]. The risk posed by a CoI depends on its nature or degree (likelihood of exerting undue influence, and the harm this could cause), which may be used to judge its implications and management for specific individuals and circumstances [5]. Together, these points are crucial to determining the appropriate management of CoI, and to understanding why the simple exclusion of all individuals or evidence with any declared conflict is often unrealistic and ill-advised.

This commentary will focus largely on the distinctions between “industry-related” and “non-industry” CoI in nutrition, for reasons that will be elaborated. These roughly align with distinctions often made between “financial” CoI and so-called “non-financial” or “intellectual” CoI. These other terms will also be used here, as they reflect common usage in literature characterizing different sources of influence. However, these terms and the presumed distinctions among them are somewhat misleading. In particular, “intellectual” and “non-financial” CoI among academics may be linked to substantial financial elements and rewards (research funding, career advancement, professional appointments, etc.).

## Industry: Not the Only Source of CoI in Nutrition

A preponderance of papers, commentaries, and analyses in the nutrition literature implicitly or explicitly limit their consideration of CoI to industry-related (“financial”) CoI [[12], [13], [14], [15], [16], [17], [18]]. The nature of the links to industry can range widely, from having a direct vested financial interest (e.g. company representatives), to commercial consultancy and advisory roles, membership in multi-partner research collaborations, or participation in professional organizations that have received industry support. Declarations of interest for publications and public positions in nutrition also focus almost exclusively on highlighting any current or past collaborations, consultancy, support, or organizational links to the industry. This orientation of public declarations is intended to facilitate transparency and CoI management. The information also feeds into a genre of academic publications that count up and highlight, but may also stigmatize individuals declaring a history of commercial funding, especially members of expert advisory committees [[12], [13], [14],^19^,^20^]. At the extreme, this can resemble a form of “scientific McCarthyism” [8] in nutrition, where what matters is “Do you now, or have you ever had, any association with the food industry?” The idea that individuals (perhaps even those authors themselves) might be conflicted in other ways is hardly considered.

Outside of nutrition, there has been much greater scholarly consideration and debate centered on CoI beyond just financial associations with industry. “Intellectual” CoI has been described as “academic activities that create the potential for an attachment to a specific point of view that could unduly affect an individual's judgment” [3,21]. The concept acknowledges that experts are inevitably influenced by their beliefs and research activities, particularly within the domain of their own publications and funding streams. Research and opinion leadership enhance professional recognition and attract tangible personal and financial rewards (opportunities for funding, travel, publicity, expert panel memberships, and career advancement) [2,6,7,22]. It is reasonable to ask, “When is a point of view a conflict of interest?” [23], but hard to deny a conflict exists when academics have established a significant record for promoting their views or advocating on topical issues. For some, this extends beyond the normal range of scholarly activities, to assertive promotion of their views through popular books and media, or fronting campaigns to advocate for policy change. This kind of activism may reflect a laudable passion, but passion is not an alternative to evidence. Such very public stances undeniably tether individuals to a particular viewpoint, with a high risk of bias on issues where there may still be substantial ongoing debate among informed experts.

The existence and potential influence of “intellectual” CoI are recognized by many major professional institutions [24]. The United States Government Office of Research Integrity advises that “If a researcher holds strong personal views on the importance of a particular area of research or set of research findings, those views should be disclosed so that others can take them into consideration…” [25]. The Cochrane Handbook refers to “…evidence of a fixated ideological or theoretical position” [26]. The Nature journals have a statement on “non-financial conflicts,” which include “institutional loyalty, personal beliefs and ambition” [27]. The International Committee of Medical Journal Editors also notes that CoI may include “personal relationships or rivalries, academic competition, and intellectual beliefs” [28].

Despite the general acknowledgment of “non-financial” or “intellectual” CoI, there exists a diversity of philosophical, ethical, and legal views on their standing and relevance [2,22,29]. Table 1 [2,6,7,14,[29], [30], [31], [32], [33]] gives a flavor of the range of views on how different types of CoI should be monitored and managed. Rodwin [2] has articulated the view that CoI in general should be better aligned to the definition used in law, and framed in terms of “incentives” (e.g., financial or other benefits) or “conflicting loyalties” (e.g., allegiance to particular viewpoints, relationships with colleagues or institutions). Grundy et al. [24], although critical of the concept of “non-financial” CoI, suggest these can be usually characterized as influences attributed to strong beliefs, predetermined views, experience, and relationships. These positionings are helpful, and suggest some specific ways of operationalizing risks of influence and bias from interests other than just industry (financial) associations.

**TABLE 1:** Examples of views on the relevance and management of industry-related or “financial” CoI vs. “non-financial” or “intellectual” CoI

Authors	Conclusions and recommendations
Millstone and Lang 2022 [14]	• Exclude those with financial CoI from expert committees • Other CoI: not considered or mentioned
Bero and Grundy, 2016 [31]; Royo-Bordonada and García-López, 2019 [30]	• Financial [industry] CoI is the real problem • Other (for example, intellectual) CoI are unavoidable and divert attention from financial CoI
Rodwin, 2018 [2]	• Distinction between financial and non-financial CoI is unhelpful • Non-financial CoI is ubiquitous and wide-ranging; no effective way to regulate, eliminate, or manage… • however, non-financial CoI may often be described and managed as a financial CoI (underlying economic self-interest)
Saver, 2012 [6]; Wiersma et al., 2018 [29]; Balon, 2022 [7]	• Non-financial CoI is similar to (or worse than) financial CoI in its impact on scientific conduct • Financial and non-financial CoI should not be treated differently
Viswanathan et al., 2014 [32]	• Non-financial CoI needs to be assessed and managed, especially on topics where the risk of influence is high • Propose a questionnaire for non-financial CoI disclosure
Cochrane handbook [33]	• Non-financial CoI should be considered, but are seldom declared and difficult to address • A “Tool for Addressing Conflicts of Interest in Trials” (TACIT) is being developed (http://tacit.one)

Abbreviation: CoI, conflict(s) of interests.

At present, any emphasis on disclosure of “non-financial” CoI and specifications for what that includes is far below what is expected for “financial” CoI [34]. “Non-financial” or “intellectual” CoI are often characterized as difficult to define and quantify and remain poorly acknowledged and rarely scrutinized. In nutrition, methods for characterizing CoI remain singularly oriented toward capturing financial associations with industry, even where other types of CoI are described [17,35]. The WHO ostensibly assesses experts’ “…financial and intellectual interests…,” to include “…any interest that may affect, or may reasonably be perceived to affect, the expert's objectivity…” [36]. Yet their actual CoI declaration asks *only* about research funding from commercial interests, and about opinions expressed in the context of official positions or testimonials. The American Society for Nutrition Guide for Authors refers to situations “…that may affect or appear to affect the impartiality and the integrity of the peer review process;” however, the publisher’s required declaration of interests is clearly oriented toward commercial support, and specifically excludes funding from public or governmental sources [37,38].

Although not in common use, there are examples of declarations specifically intended to capture “non-financial” CoI, which merit further development and use. Viswanathan et al. [32] have proposed a set of questions whereby potential expert panel members explicitly declare their record of relevant public opinions or policy advocacy, and whether they would have difficulty considering conclusions that might conflict with those prior beliefs. Asking academics to publicly declare significant sources of salary support, honoraria, and research funding on specific topics, as well as related travel funding and income from books or other media, would simply parallel what is expected for industry-related declarations. These steps to promote transparency of interests should not be controversial. They do not undermine or diminish concerns about industry-related CoI but put the full range of CoI into perspective. Expanded declarations could be facilitated by agreeing on a common and accessible reporting format and repository [39].

## Internal/“Prosocial” Compared with External/“Commercial” Influences

There are many ways in which individuals may be motivated toward biased interpretations of research results and compilations of evidence, while believing that the conclusions they draw are justified, rational, and objective [40]. Individuals may be positively biased toward accepting new information that confirms their own prior knowledge, expectations, beliefs or wishes, and to engage strategies that minimize (undervalue, suppress, and reject) less compatible information. Because “intellectual” CoI relates to the influences of one’s own beliefs and biases, it conceptually deviates from the legal foundations of CoI, which relate to the influence of third parties and divided loyalties [2]. Some commentators have therefore questioned whether one’s own internal intellectual positions can be seen as a CoI in the same way as the influences of external relationships [2,22,30].

In nutrition specifically, the food industry is widely perceived to promote scientific and policy positions favoring commerce over public health [15, 16, [41], [42], [43], [44]]. A core question is whether those wider concerns justify the application of a differential “hierarchy” of CoI in nutrition, such that links to the food industry outweigh all other potential sources of influence or bias. There is also concern that emphasizing “non-financial” CoI may have the (perhaps intentional) effect of diverting focus away from industry-related, financial CoI [30,31,45].

Against this, others have argued that the focus on commercial/financial CoI is too narrow, and “intellectual” CoI should be of equal or even greater concern [6,46]. Prevailing definitions of CoI in biomedicine do not make exceptions for how that conflict arises or its (un)avoidability. The supposed “moral high ground” may still conflict with the actual balance of evidence, and ethical or prosocial motivations do not legitimize selective suppression of alternative views [47]. Excusing or assigning less weight to CoI driven by apparent well-meaning intent implies that “white hat bias” (“distortion of information in the service of what may be perceived to be righteous ends”) [48] is acceptable.

In short, both financial and “intellectual” CoI can give rise to a risk of undue influence and bias, regardless of the presumed underlying motivations. Furthermore, the existence of a commercial relationship does not rule out acting on a prosocial motivation (belief in what is best for public health), nor does an apparent prosocial motivation rule out the influence of financial incentives (career, status, and funding).

Lastly, commercial/financial relationships have also been characterized as “avoidable” conflicts that foster a bias and sense of obligation favoring the funder [31]. In contrast, the intellectual positions of experts are seen as largely unavoidable, easily identified from public literature, and open to a balanced scientific debate [49]. However, those views do not reflect the reality of current nutrition practice, in which only industry-related CoI are consistently declared, monitored, managed, and scrutinized. Also, in many areas of food and agriculture, collaborations with industry are not only unavoidable in practice but appropriate and essential to ensure the relevance and impact of research. If (only) those individuals or research with industry-related CoI are managed by exclusion, that precludes a truly open debate and balancing of expertise and biases.

## Who Is Really Conflicted?

The preceding text outlines the broad nature of CoI and the importance of assessing the levels of risk of influence or bias introduced by secondary interests. On specific issues in nutrition, there may be some clearly identifiable commercial or intellectual stakeholders – individuals and organizations with strong incentives to adhere to and promote established positions on specific scientific issues. In other cases, secondary interests may arise through a more disinterested working relationship (e.g., researcher in a large consortium with limited industry members), with a correspondingly lower risk of bias or influence from industry-related or academic activities.

Individuals and organizations having close working relationships with the food industry may indeed have more favorable views (as well as knowledge) of commercial ingredients and practices. However, there are also many individuals and organizations that consistently advocate against the food industry in general, or specific aspects of commercial processing or ingredients. In both cases, there may be established views and intellectual leanings that raise doubts over who can be considered truly “independent” and impartial on these issues.

Consider for example the 3 nutrition scientists described in Table 2, reflecting common typologies for “hot” topics in the field. Whose views are most likely to be influenced by a secondary interest? Who is most likely to be dispassionately led by the evidence? Who should declare a CoI? By most current standards, only the “advocate” would be viewed as independent and “unconflicted” on the topic of X. Yet this individual has the greatest investment and reward in adhering to a particular view on the topic, and the most to lose (reputation, status, and funding) if that view is ultimately rejected. It may be debated whether their intellectual allegiance is considered a CoI, but it is hard to doubt their likely bias in any evaluation of X. Yet it is the views of the other 2 that are most likely to be excluded, downgraded, or attributed to a CoI. The case of the “consultant” is particularly interesting. For a CoI to exist, there must not just be a secondary interest, but one that risks biasing judgment [9]. There is an underlying presumption that payments for services to industry are *per se* an inducement to adopt and represent the corporate view. But where a consultant or advisor has been paid for their extant expert opinion, it is not obvious that their judgments have been influenced or altered by that relationship, nor have they been paid to represent or endorse those interests. The “consultant” will likely have a particular perspective on X, but so will everyone else with expertise on the topic.

**TABLE 2:** Examples of individuals with expertise in the topic “X”

**The “advocate”** “I lead a research group well-known for our work showing the adverse associations of X with diet and health. We have had substantial public funding for the topic over many years, and regularly communicate about the harms of X through scientific papers and commentaries, and social media. I am frequently sought out to attend and present my views on X at international symposia, as an invited speaker and expert panel member. **Because I have no funding from industry, I declare no conflicts of interest on the topic of X.”** **The “consultant”** “I have carried out research and reviews on X, sometimes in collaboration with scientists from the food industry. I have also accepted paid consultancies on the science of X, for companies that have commercial interests in the topic. In that role, I provide them with my expert views, update them on current and likely future developments, and offer guidance on their research and technology activities. I do not represent these companies, nor undertake or support any external lobbying, marketing, or promotional activities on their behalf. **My collaborations and consultancy services to industry are declared as a conflict of interest on the topic of X.”** **The “researcher”** “I am a research scientist whose work has included trials on the effects of X under controlled conditions. I regularly attend professional conferences, where members of our team present the results. My research sometimes challenges popular views on the relationship of X with health. Most of my work on X is publicly funded, but some projects also include participation and support from the industry. **My involvement in projects that include commercial collaborators is declared as a conflict of interest on the topic of X.”**

These points argue in favor of a more nuanced approach to characterizing and managing CoI in nutrition that takes into account the track record of the individual or organization and the nature, degree, and likely influence of their interests. In this regard, it may be helpful to distinguish between individuals or organizations whose activities are limited to generating and communicating underpinning science compared with those who actively represent or advocate for specific (scientific, political, or commercial) positions or interests. Many individuals with industry-related CoI have a meaningful track record of scholarly achievement and service to public health nutrition. They are unlikely to be shills for the food industry, despite the apparent attraction of that narrative. There are also self-proclaimed “unconflicted” academics who embellish their careers through popular books, social media, and speaking tours to promote their particular views, going beyond neutral statements of the current evidence and guidance into policy advocacy and activism.

It is clearly oversimplistic to presume that anyone linked to industry funding is intellectually dominated by those interests, whereas anyone without such associations is conflict-free and impartial. “There is nothing more partial than the pretense of impartiality” [30].

## Industry, Research, and Integrity: Judge the Science, Not the Scientists

Concerns around industry-related CoI in nutrition often overshadow due consideration of the nature or degree of the actual conflicts, the expertise of individuals, or the quality of research. At the extreme, individuals and research with industry associations are subject to good/bad “categorical thinking” [50]. They are in effect seen by some as a sort of scientific contaminant, inherently tainted regardless of nature or context [13,14,20]. This underpins a wider characterization of industry collaboration that has a number of adverse consequences for individuals, the research corpus, and evidence-based public health guidance.

First, there is an adverse effect at the level of individual researchers and research disciplines. The types of food and nutrition scientists who have collaborations with industry are likely to differ from those who do not. Industry-related CoI is inevitably more likely among researchers active in areas such as food ingredients, food technology and processing, raw materials, food safety, and sensory science. Viewed in this context, an absence of any industry-related CoI is not a sign of impartiality, but a sign of scientific expertise or activities that are less likely to attract industry investment.

There is extensive guidance available on safeguarding the integrity of research in general, as well as advised practices for collaborating with industry [18,51,52]. Nevertheless, researchers are increasingly intimidated from developing such collaborations, because of concerns their work will not be trusted, and a fear of stigmatization or exclusion from other professional opportunities such as expert panel membership [53,54]. Should individuals with a declared industry-related CoI sit on an expert advisory committee, they risk being “named-and-shamed,” and the entire committee accused of acting under the influence of “…the sticky hands of food industry” [55] if it delivers opinions that are not sufficiently antagonistic toward commercial foods and manufacturers. Yet, exclusion of those individuals creates gaps in expertise, particularly affecting experts with practical knowledge of food and ingredients.

Second, there is an adverse effect at the level of individual research publications. CoI declarations for food and nutrition journals place a heavy emphasis on any history of industry funding and collaborations. These declarations are rightly intended to provide transparency and context, although it is not actually clear how this information should be used by readers or reviewers [8]. Presumably, a declaration of industry funding should prompt a more careful look at the research and potential “spin.” If so, that sort of signaling function would be a good reason to demand equally detailed declarations of all types of interests, including “intellectual” CoI.

Declarations of industry collaboration or support have become a stigma in nutrition research publications, making them a focus of distrust and *ad hominem* criticism. Personal experience shows that such declarations on submitted manuscripts can trigger skeptical insinuations and assumptions about the integrity of authors and the validity of research, attracting comments that would be unlikely and unacceptable in ordinary academic discourse (Table 3). Many examples have been given of how research sponsors can potentially influence the conclusions and overall research corpus [30], but none of these dubious practices is exclusive to commercially supported research, and all are open to objective analysis. Short of fraud, if funding or CoI have some corrupting influence on the conduct of individual studies, that should be apparent from the protocol, design and analyses, and objective tools for quality assessment. Otherwise, if the interpretation and conclusions fairly reflect the results, it’s not clear what “funding bias” really means or how it manifests at the level of individual pieces of primary research [8].

**TABLE 3:** Examples of actual *ad hominem* comments from reviewers and editors on manuscripts declaring industry funding or employment (personal examples from the author and colleagues)

• “This would have been a stronger manuscript if the authors were not [name of company] employees, since conscious or unconscious biases may have influenced the methods or the interpretation of the data.” *(Review of manuscript with industry authors and funding)* • “We therefore can ask whether the results are really what they show... and what are the real objectives of that publication which does not apparently serve the interest of [name of company]. … We can suspect that after that "negative" study, another study will show that "added" [food ingredients] are associated with major benefits... We are waiting for that! And we will not trust such data.” *(Review of manuscript reporting ‘negative’ results of a commercial ingredient intervention)* • “…the authors …were probably given a difficult brief: to write an article that would justify the continued marketing of [type of products]” *(Editor’s rejection of a revised manuscript with 7 academic and 3 industry authors)* • “The article is clearly structured and written. I do not have any comment on the detail of the analysis. My deep concern is more general. Even clearly stated at the first page of the article, the obvious conflict of interest of the authors on this topic brings doubt about the objectivity of the work presented. More broadly, I think that this article might be a threat to the credibility of [name of journal] and more generally to the research in nutrition.” *(Review of a ‘perspective’ piece by an industry consultant)* • “There is a potential conflict of interest between the authors, all employed by a commercial company. This potential conflict of interest is emphasised by the lack of equipoise throughout the discussion and some of the conclusions.” *(Reviewer basis for rejection, no examples cited)* • “Given the funding source and employment of some of the authors I was expecting the paper to be…” *(Review of manuscript with industry authors and funding)* • “Reviewer 2 indicated [a trade organization] asked authors to write the paper and reviewed the paper prior to submission.” *(Editor’s note and query to authors. This was a complete fabrication by the reviewer; the authors fully declared their CoI, and there was no contact or involvement with any such organization.)* • “It seems that these products were all from the company where they worked, so they were chosen.” *(Review of manuscript with industry authors and funding. None of the test products were from the company where authors worked. The materials were widely available, and suppliers named in the methods.)*

Third, there is an adverse effect at the level of research aggregation for systematic reviews and expert evidence assessments, where links to industry may be deemed source of bias, independent of other, objectively defined domains [33,56]. It has also been argued that industry agenda setting and funding of research undermines the trustworthiness of nutrition science more generally [57]. Some of this rhetoric might imply that measures to suppress industry-related research and scientists are a reasonable or even advisable way to minimize, pre-empt, or redress perceived transgressions of the food industry. Indeed, a common opinion and practice is that “Industry funded studies should be clearly distinguished from those that are free from conflicts of interest” [58]. However, this deviates from best practice guidance, and risks being misused to “control [the] narrative and punish the target of censorship” [47].

The Grading of Recommendations, Assessment, Development and Evaluation (GRADE) Working Group has said: “There is no plausible rationale or supporting evidence to justify … funding bias as a separate item. In terms of conflict of interest, GRADE captures financial and non-financial interests through the existing domains for risk of bias (in particular, selective outcome reporting), indirectness, and publication bias” [59]. The Cochrane Handbook also discourages “…inclusion of conflicts of interest directly in the risk-of-bias assessment” [33]. It states furthermore that “…if trial investigators have clearly used methods that are likely to minimize bias, review authors should not judge the risk of bias for each domain higher just because the investigators happen to have conflicts of interest.” Yet this advice is routinely ignored. For example, “…to reduce the risk of bias, Nordic Nutrition Recommendations 2023 did not consider systematic reviews commissioned or sponsored by industry or organizations with a business or ideological interest” [60].

Lastly, there is an adverse effect on expert nutrition advisory committees. Much of the literature on CoI in nutrition focuses on risk assessment bodies such as the Scientific Advisory Committee on Nutrition in the United Kingdom and the Dietary Guidelines Advisory Committee in the United States [13,14,17,19,20]. Critics typically lean toward highlighting and pushing for the complete exclusion of members with any history of working with the food industry. As has been described, this reflects a very narrow view of CoI, which may serve a particular narrative but fails to allow for an assessment of the actual range and nature of the conflicts and risks they carry. In practice, this approach would also weight membership of expert committees toward epidemiology and biomedical science, to the exclusion of applied nutrition and food sciences; i.e., towards individuals with knowledge of diet and health associations, but whose actual professional expertise with food and ingredients is rather more abstract than hands-on. In reality, “Conflicts of interest are unavoidable if organizations wish to enlist a representative spectrum of experts to advise on evidence-based policies” [17]. In this light, managing CoI on advisory panels by simply replacing well-qualified experts with “…people whose key asset is [their claim to] being conflict-free” [53] is likely to be a step backward in ensuring the full range of relevant expertise is available for developing evidence-based public health guidance.

The criticisms of these expert bodies furthermore seem to presume they operate using a long-abandoned “Good Old Boys Sat Around a Table” (“GOBSAT”) approach to evidence evaluation [61,62]. There is also a tendency to overlook the important distinction between the delimited role of science experts (risk assessment) and that of policy-makers (risk management), a line that is often blurred by academics. This all ignores the movement toward evidence-based recommendations [63], and the diversity of objective tools now used to assess the quality and risk of bias of individual studies and reviews, deviations from preregistered protocols, and publication bias in the wider literature. In the best case, expert committees deploy these tools within a framework of principles and *a priori* processes for the evaluation and grading of evidence [64, 65]. The space for subjectivity and undue influence is constrained if these processes are well-defined and fit for purpose, and adherence is reinforced by disciplined oversight. Any truly meaningful critique of expert committees must identify some significant deviation or failure in following that process, not just rely on implied corruption by conflicts.

In summary, CoI and other declarations intended to provide transparency have become a basis for discriminating against individuals with industry associations and discrediting their research and views. Deselection or denigration of research based on funding alone is neither justified nor consistent with best scientific practice. There is no empirical basis for impugning, downgrading, or excluding research in a pre-emptive or punitive way, solely due to the presence of declared (industry) interests. Such practices are prejudicial and unprofessional alternatives to objective expert assessment of research activities, implying deceit or fraud that cannot be detected and need not be proven by other means [8]. Exclusion of evidence or viewpoints based solely on the presence or not of industry-related CoI subverts the use of objective tools and procedures, imbalances the range of professional expertise and perspectives, and introduces a new layer of selection bias into evidence-based public health.

### Non-industry CoI: so what?

The preceding text refers to a substantial literature focused on concerns around the influences of industry-related CoI in nutrition. In contrast, there has been little consideration of CoI unrelated to industry, nor research exploring their possible influences. Any analysis of “intellectual” or “non-financial” CoI also faces other hurdles: These are much less likely to be formally declared and lack standardized definitions and metrics [34,66].

Outside of nutrition, there is a small volume of research considering the potential influence of non-industry CoI. Miyazaki et al. [67] describe concerns around the apparent influence of “intellectual CoI” on clinical practice guidelines for hypertension. Montgomery and Weisman [68] have described a number of issues and examples relating to the influence of “non-financial” CoI in social and behavioral intervention research. Wiersma et al. [29] and Saver [6] also cite a number of concrete examples of “non-financial” CoI influencing medical research conduct and reporting. Nevertheless, these and other authors also highlight the challenge of substantiating “intellectual” or “non-financial” CoI, and weaknesses in the way this has been addressed by the expert community.

Within nutrition, analyses focused on non-industry CoI and their possible implications are almost nonexistent and largely qualitative. In their widely cited commentary on “white hat bias” in obesity research, Cope and Allison [48] highlighted examples of practices that may be “…fuelled by feelings of righteous zeal, indignation toward certain aspects of industry or other factors.” Arguments have also been made for a widespread ideological academic bias against low-energy sweeteners, evidenced by selective use and weighting of evidence, and citation bias in (largely nonindustry funded) reviews of the topic [69,70].

There are a number of studies that have reported differences in outcomes of research compared with those without declared industry interests, often framed as quantitative evidence of bias introduced by industry funding [26,71,72]. However, a difference in reported results explains little on its own, without further analysis of the research itself and alternative explanations including other possible sources of “bias” [35,70]. Moreover, this “industry bias” is not a consistent finding and is also not supported by analyses of differences in research “quality” [73,74]. The possibility that analyses of “intellectual” CoI unrelated to industry might generate similar associations has hardly been considered.

Recently, Besancon et al. [71] associated negative outcomes of research on the “Nutri-Score” labeling system with a presumed industry funding bias. However, a subsequent analysis pointed out that the balance of evidence was heavily influenced by overwhelmingly positive results reported by the academic developers of the system, relative to research independent of its developers [75,76]. This nicely illustrates the flaw in presuming that simple associations between research outcomes and funding can be reliably and causally attributed to an industry bias. They might instead (or also) reflect “bias” stemming from the intellectual allegiances of academic researchers. Based only on discrepant judgments or outcomes, it’s impossible to know who, if anyone (or everyone), is biased [40]. Rather than looking only at differences in funding, it may be more instructive to analyze associations with specific differences in research designs that may tend to generate “positive” or “negative” results.

The failure to seriously consider non-industry CoI can also lead to imbalances in expert assessments of contentious issues relating to commercial foods and ingredients. The 2023 WHO guidance on non-sugar sweeteners (NSS) was generally critical of NSS and advised against their use [77], although not without substantive expert criticism of the process and weighting of evidence [78]. A draft of the report had been reviewed by an external peer review group with “diverse perspectives” on the topic. However, most of those reviewers had an easily verifiable record suggesting likely antipathy toward NSS [[79], [80], [81], [82], [83], [84], [85], [86], [87]]. The WHO operationalization and management of CoI is primarily directed toward identifying and excluding individuals with any industry-related interests, and in practice overlooks “intellectual” CoI [36]. This rigid approach to managing CoI may guard against undue influences of industry but does not assure impartiality, and mitigates against balanced representation of the true diversity of expertise and informed perspectives.

The point here is that conflicts and biases in nutrition can come from many directions. Research and commentaries on CoI have focused heavily on the influences of industry, leading to greater scrutiny and transparency around those interests. None of this justifies indiscriminate targeting of individuals and evidence linked to the food industry, while remaining blind to other sources of conflict. Ultimately, “…when rules merely cloak an anti-industry bias in the false promise of scientific virtue, we undermine potentially productive research collaborations, dissemination of expertise, and public trust” [53].

## Summary, Conclusions, and Recommendations

Within the field of nutrition, there are important gaps and inconsistencies in the understanding, characterization, and suggested management of CoI. Most importantly, a CoI does not by itself indicate any professional impropriety, although the term has taken on pejorative connotations. A CoI can describe a wide range of activities, from relatively neutral working relationships to outright endorsement and financial dependencies. The mere existence of a CoI is not a sufficient basis for judging how the associated individuals or research evidence should be managed, without further analysis.

At present, the recognition and management of CoI in nutrition are overwhelmingly directed toward links with the commercial food industry, largely ignoring other important sources of conflict and bias. Although there is understandable concern about industry involvement in research and policy, declarations of commercial interests are open to misuse as a basis to prejudge and discredit individuals and their research, independent of other objective criteria. This includes downgrading or excluding research funded by industry, contrary to professional guidance that evidence should be judged on its content, not its creator. The stigmatization extends to expert advisory bodies and professional societies and fuels an environment where organizations are pressured to address CoI based on the risk of negative publicity, rather than an assessed risk of undue influence or bias.

Vested interests and risks of influence and bias in nutrition are clearly not limited to industry. If accepted definitions of CoI are rigorously applied, they are almost inescapable for experienced professionals and topic experts in the food and nutrition sciences. In some areas of research, collaboration with industry is essential and desired. In other areas, academics may be unconflicted in terms of commercial interests but have intellectual loyalties and financial rewards that act as inducements to adhere to particular views. Individuals may have a very prominent record of opinions and advocacy on specific issues, with substantial benefits for their funding, professional status, and career opportunities.

Individual CoI can therefore only be realistically and fairly managed by assessing the nature and degree of conflicts, and their potential impact. This requires full disclosures that allow for a fair and meaningful assessment of risks, and that is not possible if only a subset of secondary interests is considered. There will always be justifiable grounds for excluding individuals and organizations from involvement in topics where they have a clearly established loyalty and an entrenched position. This applies equally to commercial and intellectual “stakeholders,” whose goals, reputations, and revenue streams root them to a particular view. Managing (only) industry-related CoI by categorical exclusion of individuals and evidence has a number of adverse and unjustified impacts. Although seeking to prevent undue influences of “industry actors,” it undermines objective methods of evidence assessment and distorts the balanced representation of the true range of professional expertise and perspectives on contemporary issues in public health nutrition.

Based on these considerations, a number of specific recommendations may be advised. There is a fundamental need for a more professional and consistent understanding and balanced application of the concept of CoI in our field. It should be accepted that CoI is prevalent and often unavoidable for senior academics and other professionals in food and nutrition, is not *per se* a sign of compromised integrity, and is clearly not limited to associations with industry. Given this, the procedures for characterizing and managing CoI for research and public appointments must be proportional and fair and applied in a way that preserves a balanced representation of authoritative expertise and views. Declarations of non-industry (‘non-financial” or “intellectual”) interests should be explicitly prescribed, paralleling the expectations and significance attached to industry-related or “financial” CoI. On specific issues, a CoI should be recognized when individuals have a manifest allegiance to or significant professional stake in a particular intellectual or policy position, regardless of their funding sources, alliances, or presumed motivations. Ultimately, concerns about CoI cannot reliably be based simply on how they “appear,” but only on a meaningful assessment of the risk posed. The practice of judging and discrediting individuals and research purely on the basis of declared industry-related interests should therefore be discouraged, both within the field and by outside observers. Greater emphasis and reliance should be placed on the availability and application of objective tools and procedures to assess and manage the risks posed by CoI and bias in research and the development of evidence-based recommendations.

## Author contributions

The sole author was responsible for all aspects of this manuscript.

## Conflict of interest

DJM is a retired academic and industry research scientist. He has not received any support or incentives in any form for work on this topic. He is a shareholder and was until 2019 an employee in Unilever, and has since provided independent scientific consultancy in the areas of sugars and sweeteners for Unilever, Cargill Inc., Danone, CBC Israel, and Tate & Lyle PLC. He is an advisor to a number of academic research projects, and a member of the UK Scientific Advisory Committee on Nutrition, which maintains a full list of his declared interests. Neither of these nor any other organization had any role in the conception or preparation of this manuscript, and the views expressed here are solely those of the author.
